# The causes and consequences of frailty: A comparative cohort study in Costa Rica and the United States

**DOI:** 10.1093/geroni/igaf122.2380

**Published:** 2025-12-31

**Authors:** Amanda Lehning, Carolina Santamaria-Ulloa, Ericka Mendez-Chacon, Jihyeong Jeong

**Affiliations:** University of Maryland Baltimore, Baltimore, Maryland, United States; University of Costa Rica, San Pedro de Montes de Oca, San Jose, Costa Rica; University of Costa Rica, San Pedro de Montes de Oca, San Jose, Costa Rica; University of Maryland Baltimore, Baltimore, Maryland, United States

## Abstract

Frailty is a common condition among older adults that results from aging-related declines in multiple systems and increases vulnerability to negative health outcomes. The aim of this study is to compare the causes and consequences of frailty among older adults from Costa Rica and the United States. Using data from community-dwelling older adults from the Costa Rican Longevity and Healthy Aging Study (CRELES, n = 1,790) and the National Health & Aging Trends Study (NHATS, n = 6,680), first we estimated Cox proportional hazard models to examine the association between frailty and all-cause mortality 8 years later. The death hazard for frail compared to non-frail older adults was three-fold in Costa Rica (HR = 3.14, p < 0.001) and four-fold in the US (HR = 4.02, p < 0.001). At baseline 21.9% of NHATS respondents met the criteria for frail compared to 16.2% of CRELES respondents. Second, we estimated multinomial regression models, stratified by sex, to examine frailty transitions (i.e., worse, same, or better) between two waves. Among men in the Costa Rican sample, respondents who were older had higher odds of becoming more frail; those who reported lower income, had fewer years of education, or had been diagnosed with arthritis had higher odds of becoming less frail. In the United States, however, there were no significant associations between sociodemographic characteristics, health and functioning, and frailty transitions except for diabetes/hypertension. Findings indicate differential pathways to frailty between these two countries, and indicate that social, environmental, and policy influences should be examined in future research.

